# Supplementary material for: Content-rich biological network constructed by mining PubMed abstracts
Source: BMC Bioinformatics. 2004 Oct 8;5:147. doi: 10.1186/1471-2105-5-147 (PMC528731; doi:10.1186/1471-2105-5-147)
Supplement: Additional File 2 — The original results of the above study (non-essential files are deleted to keep the file size under the limit set by BMC bioinformatics). [file 1471-2105-5-147-S2.bz2 › chilibotAdditionalFile2/dip10/717ID10781811E18054/html/TATA_TATB.html]

 


 **TATA** and **TATB** 
  
Found 20 abstracts in PubMed, retrieved 10.  
 

 What does Google say? 
 PDF only 
| .edu only 

---

**Interactive relationship** (e.g. stimulation, inhibition, etc)

**Neutral relationship**- The three isolated, affinity tagged Tat  [ **TATA** ]  complexes comprised  **TatA** ,  **TatB**  and TatC in each case, demonstrating a strong interaction between these three subunits.  Ref: 12823967 J Mol Biol, 2003
- A chimeric protein comprising the N terminal region of  **TatA**  fused to the amphipathic and C terminal domains of  **TatB**  supports a low level of Tat  [ **TATA** ]  activity in a strain in which the wild type copy of either  **tatA**  or  **tatB**  but not both is deleted.  Ref: 12374820 J Bacteriol, 2002
- Surprisingly, large scale mutagenesis of the conserved amphipathic regions of  **TatA**  and  **TatB**  leads only to minor effects on Tat  [ **TATA** ]  dependent export suggesting that this particular feature is not central to the translocation mechanism.  Ref: 12606028 FEBS Lett, 2003
- Its main components are  **TatA** ,  **TatB**  and TatC, all of which are required for Tat  [ **TATA** ]  dependent export.  Ref: 12773183 Biochem Soc Trans, 2003
- Proteins bearing a signal peptide with a consensus twin arginine motif are translocated via the Tat  [ **TATA** ]  pathway, a multiprotein system consisting minimally of the integral inner membrane proteins  **TatA** ,  **TatB** , and TatC.  Ref: 12427031 Biochemistry, 2002
- Our data suggest that the  **TatA** ,  **TatB**  and TatC proteins are found in at least two major types of high molecular mass complex in detergent solution, one consisting predominantly of  **TatA**  but with a small quantity of  **TatB** , and the other based on a TatBC unit but also containing some  **TatA**  protein.  Ref: 12367533 J Mol Biol, 2002

**Non-interactive relationship** (e.g. studied together, co-existance, homology, etc.)

- The Escherichia coli twin arginine translocase conserved residues of  **TatA**  and  **TatB**  family components involved in protein transport.  Ref: 12650927 FEBS Lett, 2003
- Truncation analysis of  **TatA**  and  **TatB**  defines the minimal functional units required for protein translocation.  Ref: 12374820 J Bacteriol, 2002
- These results imply that the transmembrane and amphipathic helical regions of  **TatA**  and  **TatB**  are critical for their function but that the C terminal domains are not essential for Tat  [ **TATA** ]  transport activity.  Ref: 12374820 J Bacteriol, 2002
- The  **TatA**  and  **TatB**  proteins are essential components of the twin arginine protein translocation pathway in Escherichia coli.  Ref: 12374820 J Bacteriol, 2002
- Four insertions analyzed had no effect on the stability of the altered TatC proteins or on membrane assembly of the  **TatA**  and  **TatB**  proteins.  Ref: 12163163 FEBS Lett, 2002
- Here is the report of knocked out the  **tatA** ,  **tatB**  and tatC genes of the V. cholerae by suicide plasmid homologous recombination technology.  Ref: 12561481 Yi Chuan Xue Bao, 2002
- The first region is the interdomain hinge between the transmembrane and the amphipathic alpha helices of  **TatA**  and  **TatB**  proteins.  Ref: 12650927 FEBS Lett, 2003
- In this report we have studied the functions of conserved residues within the structurally related  **TatA**  and  **TatB**  proteins.  Ref: 12650927 FEBS Lett, 2003
- The second region is within the amphipathic helices of  **TatA**  and  **TatB** .  Ref: 12650927 FEBS Lett, 2003
- The integral membrane proteins  **TatA** ,  **TatB**  and TatC are essential components of this pathway.  Ref: 12367533 J Mol Biol, 2002
